# Supplementary material for: Are carbon emissions trading and green financial instruments synergistic? -Comprehensive quantitative research based on content analysis
Source: PLoS One. 2024 Mar 7;19(3):e0298601. doi: 10.1371/journal.pone.0298601 (PMC10919601; doi:10.1371/journal.pone.0298601)
Supplement: S1 Data — (ZIP) [file pone.0298601.s001.zip › Data/地级市Z_F工作报告.zip!/╕╩╦α/╜≡▓2╩╨/└┤╫╘╣1⁄2╓┌║┼í╛┬φ┐╦╩2╛▌═°í┐_2014.docx]

各位代表：
    现在，我代表市人民政府向大会作工作报告，请予审议，并请各位政协委员和列席会议的同志提出意见。
    **一、2013年工作回顾**
    2013年，面对经济下行压力加大、主导工业产品价格下跌、财政收入和企业效益下滑的不利形势，在省委、省政府和市委的坚强领导下，我们深入实施“1234”发展战略，在改革开放中前进，在真抓实干中发展，在应对考验中提升，继续保持了经济平稳发展和社会和谐稳定，基本完成了市七届人大二次会议确定的各项目标任务。预计全年完成地方生产总值250.5亿元，同比增长15.1%，其中第一产业增长6%，第二产业增长16.5%，第三产业增长11%；固定资产投资242亿元，增长31%；社会消费品零售总额58.6亿元，增长15%；大口径财政收入34.7亿元，下降6.9%；地方公共财政收入15.83亿元，增长18.8%；进出口贸易总额39亿美元，增长15%；城镇居民人均可支配收入26323元，增长13%；农民人均纯收入9147元，增长16%；人口自然增长率4.88‰。
    项目建设成效显著。坚持把贯彻落实“3341”项目工程作为转型跨越、富民兴市的重要抓手，深入开展“三个千亿元”活动，既促进了经济增长，又增强了发展后劲。95个重点项目开工率达到85%，金武高速公路建成通车，市区2&times;330兆瓦热电联产实现单投，330千伏上河湾变电站、金阿高速公路等项目进展顺利，石羊河流域重点治理、东河灌区续建配套与节水改造、国家重点公益林管护等项目完成年度任务。招商引资到位资金181.7亿元，同比增长88.5%；第十九届兰洽会暨民企陇上行签约项目开工率达到72%，资金到位率20%，位居全省前列。谋划储备500万元以上项目420个，概算总投资1100亿元，“引大济西”工程规划报告已报国家水利部，金川集团公司3&times;35万千瓦自备发电机组、法国玛努尔高端装备铸锻件、西藏城投1万吨碳酸锂提纯等项目前期工作取得积极进展。
    工业结构更趋优化。及时制定促进经济稳定增长、加快开发区发展、推动非公经济跨越发展、保障工业循环经济上下游企业有机衔接等一系列政策措施，扎实开展“抓管理、降成本、增效益”活动，三大重点产业加快发展，多点支撑、多元发展、多极增长的经济发展新格局加速形成，新增规模以上工业企业21家，预计规模以上工业增加值达到156亿元，同比增长17% 。30万吨铜材深加工、单晶铜键合引线等项目顺利建设，350万只圆筒印花镍网建成，镍网生产能力占全国的40%，我市成为全国最大的镍网生产基地。西坡、金武公路、河清滩三个百万千瓦级光伏发电场初具规模，3万吨太阳能光伏支架一期、5万吨支架镀锌一期等项目建成投产，振发新能源光伏产业园、艾力克光伏发电组件等项目加紧建设，光伏发电并网容量达到143.5万千瓦，我市成为全省首个百万千瓦级光电基地。10万吨无机纤维、40万吨离子膜烧碱二期、30万吨PVC、1万吨白烟灰综合利用、16万吨干法乙炔等项目加快建设，150万吨捣固焦、20万吨合成氨等项目建成投产，我市被列为全国首批循环经济示范市。
    “三农”工作扎实推进。农业结构调整成效明显，特色优势作物占农作物播种面积的93%，新改建日光温室1239座、食用菌棚1012座、塑料拱棚5120座，新建标准化养殖小区20个、万只羊场4个、千头牛场2个。新型农业经营主体发展迅速，申报认定和注册家庭农场30个，农民专业合作社达351个。现代农业示范园区初具规模，“一区三园两基地”加快建设，示范带动作用和产业集聚功能日益凸显，土地流转率达到31%，农机作业综合水平78%，秸秆综合利用率75%，废旧农膜回收利用率68%，规模畜禽养殖废弃物利用率80%。农业标准化生产扎实推进，双湾食葵被认证为地理标志农产品，全市“三品一标”覆盖率达到75%。多渠道输转城乡富余劳动力9.02万人，实现劳务收入11.03亿元。“双联”行动和扶贫开发融合发展，《关于进一步支持永昌县加快发展的意见》和《永昌县扶贫开发三年计划》出台实施，筹集双联帮扶基金1194万元、救助基金456万元，帮助建设强村富民项目123个，全市贫困人口减少9000人，贫困面下降3.71个百分点。
    第三产业快速发展。太西煤物流中心、惠博物流仓储中心加快建设，金三角商厦改扩建、金川区物流配载信息中心建成投用，北京路大市场紫晶广场、天庆惠民综合市场完成主体工程，改造提升15个村级商贸综合服务社，商贸流通业发展基础不断夯实。成功举办第二届骊靬文化国际旅游节和2013国际青少年生存训练营活动，骊靬文化产业园加快建设，电影《罗西与莫妮卡之骊靬情》在国内院线上映，评书广播剧《罗马军团消失之谜》在中央人民广播电台播出，骊靬文化知名度和影响力不断提升。金昌大剧院建成，市图书馆、市博物馆和县文化中心、区三馆完成主体工程，市传媒中心开工建设，新建7个乡镇综合文化站，覆盖城乡的公共文化服务体系不断完善，我市被命名为首批国家公共文化服务体系示范区。
    城市功能日益完善。城乡规划体系日臻完善，城乡综合交通、中心城区环卫设施等专项规划，龙首新区等控制性详细规划，以及金水新区街景绿化、龙泉景观带三期、龙首湖公园等景观规划编制完成。新区建设快速推进，八冶科技大厦、金昌商会大厦建成投用，市限价商品房、龙首湖公园、龙泉景观带二期以及市区三条主干道路街景风貌改造等项目扎实推进，金昌火车站改造工程、杭州路、西坡光伏大道等4条城市道路和金川路过街通道建成投用，金昌机场开通北京、嘉峪关航线。城中村改造和小城镇建设步伐加快，天庆家园高崖子新村全面建成，西坡城中村改造二期顺利推进，市区、县城以及河西堡、朱王堡等重点小城镇在聚集农村二三产业、转移农村富余劳动力、带动农村经济社会发展等方面功能进一步增强，预计全市城镇化率达到66.39%。城市管理水平进一步提高，国家环保模范城市创建工作稳步推进，全国城市文明程度指数测评位列61个地级提名资格城市第19位，未成年人思想道德建设工作测评位列参评地级城市第16位，双拥模范城“七连冠”届中检查评估位居全省前列，国家园林城市成功创建，市区环境空气质量优良天数达到321天，我市进入全国宜居城市百强行列。
    社会事业全面进步。自主创新能力明显提升，建立4个科技研发中心，28个项目列入省级和国家级科技专项，培育农业科技示范点10个，申请专利380 件。教育事业优先发展，冶金学院2号教学楼、永昌七中建成投用，理工中专迁建、永昌职中实训楼、14所幼儿园新改建等项目进展顺利，高中阶段毛入学率和义务教育巩固率分别达到99.65%和99.87%，高考二本以上上线率达到43.39%，位居全省前列。医药卫生体制改革有序推进，公立医院管理体制改革进一步深化，基本公共卫生服务均等化水平不断提高，市人民医院和市第一人民医院迁建项目开工建设，5所乡镇卫生院附属用房等项目进展顺利。全民健身运动广泛开展，建成“一村一场”50个、体育惠民工程7个、乡镇农民健身中心4个、城市社区高标准健身场所2个。应急预案体系进一步健全完善，应急预案演练切实加强，应对突发公共事件能力不断提升。统计、审计、工商、质监、气象、档案、残疾人、地方志、妇女儿童、国土资源、防震减灾、民族宗教、外事侨务和住房公积金等社会事业都取得了新进步。
    民生福祉持续改善。新增城镇就业岗位2.59万个，发放小额担保贷款1.55亿元。启动实施大病保险试点工作，取消居民医疗保险基金和大病补助基金最高报销支付限额，三级、二级医疗机构住院报销比例分别由60%和70%提高到75%和80%；城市低保标准由月人均266元提高到306元，农村低保标准由年人均1488元提高到1907元，农村五保供养标准由每年4300元提高到4500元，企业职工和“五七工、家属工”月分别增加基本养老金126元和85元；60岁以上城乡老人免费乘坐公交车，高龄补贴范围从90岁以上扩大到80岁以上。农村计划生育家庭奖励扶助和独生子女伤残家庭、死亡家庭特别扶助标准年人均分别增加240元、1080元和1980元。4800套保障性住房基本建成，改造农村危旧房1700户。社会管理创新“六大行动计划”深入实施，社会稳定风险评估工作基本实现全覆盖，社会治安防控体系持续完善，“六五”普法工作不断加强，“平安金昌”建设深入推进，人民群众安全感和幸福指数明显提升。
    改革开放深入推进。金川集团公司分离办社会职能、金泥集团公司股份制改革稳步推进，三洋金源农牧公司进入上市辅导期。公共资源交易中心挂牌运行，食药监管体制改革全面完成，工商、质监移交地方管理，农村综合配套改革进一步深化，营业税改征增值税试点、农村土地承包经营权登记试点、集体林权制度配套改革和水利改革试点等工作有序推进。充分发挥财税职能作用，扶持发展骨干税源，积极培植新型税源，严肃查处偷、逃、抗税行为，征管工作进一步加强。牢固树立过“紧日子”的思想，着力优化财政支出结构，优先安排保运转、保民生、保重点的支出，资金使用效益不断提高。首届循环经济国际博览会成功举办，海关及出入境检验检疫局办公楼主体完工，甘肃银行金昌天津路支行挂牌运营，新增小额贷款公司6家、融资性担保公司3家。积极搭建银企对接平台，与3家省级金融机构签订战略合作协议，驻地金融机构为216个项目授信352.4亿元，落实贷款176.2亿元，金融支持经济社会发展力度明显加大。高标准完成舟曲灾后援建任务，对口支援天祝藏区工作成效显著。
    政府效能明显提升。自觉接受人大工作监督、法律监督和政协民主监督，积极听取民主党派、工商联、无党派人士和人民团体意见，人大代表议案、建议和政协委员提案办结率达到100%。狠抓党风廉政建设，深入开展“效能风暴”行动，市政府政务服务中心、开发区综合服务中心建成启用，公共资源交易活动实现了统一交易、统一管理、统一监督。修订完善政府工作规则和“六大考核激励机制”，清理行政审批事项433项，取消84项，减幅20%；下放8项，精简16%，政府行为进一步规范，办事效率进一步提高，机关作风进一步好转。严格执行中央和省、市委改进工作作风、密切联系群众的有关规定，下功夫清理和规范各种达标、评比、表彰以及论坛、庆典等活动，会议数量同比减少45%，市级“三公经费”下降20.5%。完成机关办公用房和楼堂馆所建设项目清理工作，县级以上领导干部办公室整改到位，公车编制、使用范围进一步规范。
    各位代表，刚刚过去的一年，是我们应对各种挑战、经受重大考验的一年，取得的成绩实属不易。这是省委、省政府和市委科学决策、正确领导的结果，是市人大、市政协监督支持、共识共为的结果，是地企、军地密切配合、通力协作的结果，是全市人民艰苦奋斗、和谐进取的结果，是历届班子坚持不懈、奋发努力打下坚实基础的结果。在此，我代表市人民政府，向过去一年所有为金昌改革和发展作出贡献的各族各界人士，表示衷心的感谢，并致以崇高的敬意！
在看到成绩的同时，我们也清醒的认识到，经济社会发展和政府工作还存在不少困难和问题：一是农业基础和农村发展条件依然薄弱，贫困区域居住条件差，贫困居民脱贫难度大，统筹城乡发展和区域发展的任务重。二是经济增长下行压力加大，循环经济产业链不稳，主导工业产品价格下跌，工业企业效益下滑、发展困难增多。三是经济结构不尽合理，一、三产业比重偏低，中小微企业和非公企业发展缓慢。四是经济增长内生动力不足，自主创新能力不强，推动科技创新、培育新的经济增长点还需下更大功夫。五是社会创新管理难度加大、解决好关系群众切身利益的收入分配、教育医疗、就业社保等方面问题任务艰巨。六是工业增加值、固定资产投资、社会消费品零售总额、城镇居民人均可支配收入等四项指标未完成省政府下达的目标任务。七是政府职能转变还不到位，一些工作人员责任意识、服务意识不强，工作效率不高的问题依然存在，作风建设和廉政建设需要进一步加强，等等。我们一定要牢记职责使命，加倍努力工作，采取切实有效的措施解决好这些问题，决不辜负省委、省政府和市委的期望，决不辜负人大代表、政协委员的信任，决不辜负全市人民的重托！
    **二、2014年主要工作**
    各位代表，今年是贯彻落实党的十八届三中全会精神的开局之年，也是实施“十二五”规划承前启后的关键一年。做好今年的政府工作，对于全面深化改革，率先全面建成小康社会，具有十分重要的意义。
    当前，国际经济形势依然错综复杂、充满变数，世界经济低速增长态势仍将延续，我国经济正处于增长速度换档期、结构调整阵痛期和前期刺激政策消化期叠加阶段，我省经济正处于经济发展黄金期、政策机遇叠加期和后发优势释放期。对我们这样一个产业结构单一、经济发展资源依存度高、外向型经济特征明显的工矿城市而言，要保持经济平稳健康发展，挑战与机遇并存。党的十八届三中全会发出了全面深化改革的动员令，《中共中央关于全面深化改革若干重大问题的决定》是我们迈向改革开放新征程的科学指南和行动纲领；国家打造“丝绸之路经济带”的战略构想，进一步加大对中西部地区特别是贫困地区的支持力度，对西部地区实施差别化的区域经济政策等，为我们提供了千载难逢的政策叠加机遇；省委、省政府描绘了建设幸福美好新甘肃的光明前景，着力实施“3341”项目工程和“1236”扶贫攻坚行动，为我们指明了做好工作的着力重点和行动方向。经过不懈努力，我市优势产业和基础设施建设明显加强，园区经济、重点产业等新的增长点正在加速形成，特别是通过深入学习习总书记系列重要讲话精神，全市干部群众推动加快发展、转型跨越的思想更加统一、措施更加有力、干劲更加充足。我们坚信，只要敢于直面困难矛盾，善于用足用活政策，勇于深化改革开放，变压力为动力，化挑战为机遇，金昌就一定能够在新一轮改革发展中赢得主动、赢得优势、赢得胜利！
    今年政府工作的总体要求是：全面贯彻落实党的十八大和十八届二中、三中全会精神，按照省委十二届六次、市委七届八次全委扩大会的工作部署，围绕把我市打造成丝绸之路经济带资源加工基地、全国循环经济示范市、我省向西开放开发重要平台，紧扣“1234”发展战略，坚持稳中求进、改革创新，全面深化改革开放，切实保障改善民生，奋力推进经济、政治、文化、社会和生态文明建设，努力建设繁荣文明和谐宜居现代金昌，为率先在全省全面建成小康社会奠定坚实基础。
    综合分析国家政策导向和我市发展环境、结构特征，按照“十二五”规划和全面建成小康社会的目标要求，2014年经济社会发展主要目标是：生产总值增长11%；规模以上工业增加值增长13%；固定资产投资增长26%；大口径财政收入增长10%；地方公共财政收入增长8%；社会消费品零售总额增长11%；城镇居民人均可支配收入增长11%；农民人均纯收入增长13.5%；居民消费价格指数涨幅控制在3.5%左右；万元GDP能耗下降2.5%以上；城镇登记失业率控制在4%以内；人口自然增长率控制在7‰以内。
    围绕上述目标，重点做好以下八个方面的工作。
    （一）着力加强项目建设，切实增强发展后劲。 坚持把项目带动行动作为稳增长、调结构、增后劲的重要载体，深入实施“3341”项目工程，进一步突出重点、明确责任、加快推进。
    扎实开展“千亿元”项目大谋划活动。抢抓我市被列为全国循环经济示范市、被纳入老工业基地调整改造规划、资源型城市可持续发展规划、园区循环化改造范围等政策叠加机遇，突出政策转化应用，加强向上衔接、企业对接、部门联结，精心谋划生成和梯次开发培育一批符合资源禀赋、适应市场需求、壮大经济规模的特色产业项目和一批打基础、利长远、惠民生的基础设施项目，确保“3341”工程项目库项目储备规模达到1200亿元以上。重点做好兰州至张掖铁路新增三四线、金山高等级公路、750千伏输变电2号主变、有色金属产品原料交易市场、化工产品集散中心等项目论证争取工作，加快推进“引大济西”、超细金属及氧化物、多晶硅切片、金川集团公司3&times;35万千瓦自备发电机组、韩国奥瑟亚株式会社新能源开发等项目前期工作，提高项目的成熟度和可行性，争取早日开工建设。
    扎实开展“千亿元”项目大招商活动。按照“产业园区化、技术高端化、生产低碳化”的思路，着眼资源代价、社会就业、财税贡献、投资强度和带动功能，紧盯世界500强，聚焦中国500强，瞄准中亚五国，锁定发达地区，坚持引资、引技、引智有机结合，坚持地企联合招商、节会招商、以商招商、产业招商、园区招商、全民招商多措并举，着力引进一批高技术、高效益、低能耗、低排放、延伸产业链、提高关联度的重大龙头项目，形成工业向园区集中、产业向园区集聚、企业向园区集结、资源向园区集约、资本向园区集流的新型工业化模式。继续强化招商引资目标管理责任制，确保招商引资签约规模达到1000亿元以上，项目落地率50%以上，资金到位率20%以上。
    扎实开展“千亿元”项目大建设活动。突出重点产业、重大基础设施、重要民生项目，全年组织实施各类投资项目309个，总投资近1000亿元，其中重点项目103个，总投资784亿元；生产经营性项目230个，占项目总数的74.4%；续建124个，总投资484亿元；新建185个，总投资508亿元。续建和新建项目中，1-5亿元80个，5-10亿元30个，过10亿元24个。继续落实市级领导包抓责任制和招商引资签约项目联络员制，强化协调服务和用地、资金、配套设施等项目实施要素保障，推动项目建设与土地开发、盘活资产、引进社会资本一体运作，推进质量、效益、安全、环保、廉政“五同步”，加快项目建设进度，完成固定资产投资305亿元。
    （二）着力发展循环经济，切实加快新型工业化进程。积极适应国家化解过剩产能的新要求，紧紧围绕“一区三园”发展壮大，大力实施“五个千万级工程”，加快推进以科技含量高、经济效益好、资源消耗低、环境污染少、人力资源优势得到充分发挥为特征的新型工业化发展，促进产业向高端化、产品向终端化方向发展。 
    大力实施千万吨有色金属矿产资源加工工程。积极支持金川建设产业园和再生资源工业园建设，重点实施硫化铜贫矿资源高效开发、单晶铜键合引线、圆筒印花镍网三四期等项目，开工建设1500吨特种高温合金材料、50万吨高端装备铸锻件等项目，加快建设金川东部贫矿开采等项目，30万吨铜材深加工、10万吨无机纤维等项目建成投产。
    大力实施千万吨工业固废综合利用工程。加快全国工业固废综合利用示范基地建设，重点实施镍铜矿伴生铂族金属高效回收与综合利用、5000吨电石渣水泥熟料资源综合利用、镍阳极泥综合利用、铜阳极泥稀贵金属综合回收、危废资源化处置中心、磷石膏资源化利用等项目，积极推进冶炼炉渣综合利用项目，开工建设中间含铜物料综合利用等项目。
    大力实施千万吨化工循环产业建设工程。重点支持金川集团公司实施40万吨离子膜烧碱二期、30万吨PVC等项目，着力实施60万吨硫基复合肥等项目，开工建设1万吨碳酸锂提纯、6万吨三氯化铁、20万吨硝铵等项目，16万吨干法乙炔、8万吨液化甲烷、5万吨磷酸一铵、5万吨轻质碳酸钙等项目建成投产。
    大力实施千万吨煤炭资源深加工工程。坚持依煤而电、依煤而化、依煤而冶，积极承接内蒙古和新疆煤炭以及中亚地区煤气资源加工利用，积极推进金川集团公司褐煤分质利用、韩国奥瑟亚株式会社30万吨煤焦油深加工等项目，加快实施市区2&times;330兆瓦热电联产、三毅10万吨粉煤灰综合利用等项目，日升隆60万吨煤低温干馏、予能煤业60万吨精选煤等项目建成投产。
    大力实施千万千瓦级风光电资源开发工程。加快推进三个百万千瓦级光伏发电场，以及上海电气风电设备制造基地、振发新能源光伏产业园500兆瓦跟踪式光伏支架等项目建设，力争风光电装机规模达到200万千瓦，我市被列为全国新能源示范城市。开工建设西大河北总干渠一、二级水电站等项目，全面建成330千伏上河湾变电站、海诺尔生活垃圾焚烧发电一期工程等项目。
    （三）着力夯实农业基础，切实提高农业和农村经济发展质量。把保供增收惠民生、扶贫致富奔小康作为重要任务，进一步强化农业、惠及农村、富裕农民。
    实施美丽乡村建设计划。按照“一村一景、一村一貌、一村一品、一村一韵”的要求，实施“十村示范百村整治”工程，合理定位村庄主题特色，统筹路网、林网、供水网、电网、通讯管网、垃圾处理网等一体化建设，大力改善农村人居环境，把全市广大农村建设成“村庄秀美、环境优美、生活甜美、社会和美”的宜居宜业宜游幸福美好新农村。把示范村建设、空壳村整治、贫困村搬迁和鼓励农村居民进城落户结合起来，通过财政资金引导、项目资金整合、社会资金参与、工商资本支持和金融资本扶持等有效途径，规划建设50户以上集中居住示范点20个，新建居住小区2个、农村社区5个，搬迁改造农宅5000户，改造农村危旧房4000户，新建通村公路60公里，努力形成以工促农、以城带乡、工农互惠、城乡一体的新型工农城乡关系，让广大农民平等参与现代化进程、共同分享现代化成果。
    实施循环农业促进计划。着力加强清河现代农业循环产业园、金昌现代畜牧循环产业园、金川现代循环农业示范园建设，大力发展节水、绿色、生态、立体、循环生态农业，秸秆综合利用率达到80%，废旧农膜回收利用率达到70%，规模畜禽养殖废弃物利用率达到85%。认真落实石羊河流域灌溉面积压减任务，重点实施石羊河流域重点治理、新增千亿斤粮食产能工程、东河灌区续建配套与节水改造、金川河河道治理、东大河渠首泄洪闸除险加固等项目，推广垄作沟灌33万亩、玉米全膜沟播14万亩、膜下滴灌5.4万亩，治理河道12公里。启动实施祁连山水源涵养区生态环境保护和综合治理、生态功能县、沙化土地封禁保护补助试点县等项目，完成人工造林4800亩、封山育林2.3万亩，沙化土地综合治理15万亩。   
    实施富民产业培育计划。围绕做大基地、做强龙头，着力培育专业合作社、农业企业、家庭农场“三大经营主体”，大力发展农业示范园区、农产品流通市场和特色产业专业镇村“三大发展载体”，切实强化结构调整、科技推广、质量安全“三大保障手段”，重点实施菜篮子、冷链物流、者来寨酒业万吨酒庄、三洋肉食品加工、天牧乳业万头牛场等建设项目，培育专业合作社70家，新增市级以上龙头企业8家，扶持家庭农场20家，建立“三品一标”示范基地10万亩，农产品质量抽检合格率达99%以上，不断提升农业标准化、规模化、产业化、特色化、品牌化发展水平。
    实施农民收入倍增计划。正确处理粮食生产安全与农业结构调整的关系，稳定粮食播种面积，大力发展优质肉羊、优质肉牛、无公害蔬菜、啤酒大麦、现代制种等特色产业，新建标准化养殖小区20个、万只羊场2个，增加经营性收入；结合“农村劳动力转移培训计划”、“绿色农业培训工程”等项目，以务工技能为重点培训内容，以职教城为重要培训基地，以就近转移为主要输转方式，输转富余劳动力8万人（次），实现劳务收入11亿元，增加工资性收入；按照建设城乡统一用地市场的改革取向和赋予农民更多财产权的政策要求，稳妥推动农村资产“确权赋能”，组建县、区农村产权交易市场，土地流转率达36%以上，提高农民在土地增值中的分配比例，增加财产性收入；认真落实各项支农惠农富农政策，增加转移性收入，确保农民人均纯收入突破万元大关。
    实施扶贫开发三年计划。深入实施“1236”扶贫攻坚行动和“双联”行动，切实走好高效节水、规模经营、设施种植、草食畜牧业、移民搬迁、劳务输转、发展第三产业等“七条路子”，大力实施村镇建设和居民安居、村容村貌整治、道路畅通、饮水安全和农田水利、农网升级、能源生态等“六大工程”，新建维修渠道100公里，新建改造通社公路120公里，新建食用菌棚1000座、日光温室1000座、塑料钢架拱棚1500座、养殖暖棚4500座，发展高原无公害蔬菜1.5万亩，推广膜下滴灌1.4万亩，输转贫困村劳动力4500人（次），完成易地扶贫搬迁104户328人，确保扶贫对象人均纯收入增幅高于全市平均水平2个百分点以上。积极争取将永昌县列为全省国开行贷款支持范围，大力推进整村扶贫开发，加快建设东达蒙古王集团永昌县扶贫开发基地，发展獭兔养殖1000户。
    （四）着力发展现代服务业，切实提升第三产业发展水平。牢牢把握扩大内需战略基点，坚持生产性服务业和生活性服务业双轮驱动、传统服务业和现代服务业齐头并进，努力促进第三产业发展提速、比重提高、水平提升。
    加快商贸物流业发展。大力改造提升城乡消费设施和服务体系，加快盘旋路商业综合体、国芳万和城、金戈市场改造、永昌万祥城二期和建材汽修市场等项目建设，金三角市场改扩建工程、北京路大市场紫晶广场建成投入使用。改造提升农贸市场 2个、村级商贸综合服务社19个，畅通农产品进城、工业品下乡渠道。全力创建国家“智慧城市”，加强4G网络信息基础设施建设，促进信息消费升级。突出抓好太西煤物流中心、金昌发电公司煤炭储备中心、惠博物流仓储中心、祥泰物流中心等项目。积极发展第三方物流，鼓励大中型企业剥离物流业务，促进物流处理向物流代理转变，企业物流向物流企业转变，传统物流向现代物流转变。
    加快文化旅游业发展。围绕华夏文明传承创新区建设，巩固提升国家公共文化服务体系示范区创建成果，抓紧编制文化产业发展规划，抓好金昌文化创意产业园、骊靬文化产业园、华轩欢乐谷甘肃金水湾国际青少年夏令营基地、市传媒中心、市博物馆、金昌文化街、西路军永昌保卫战纪念馆等项目建设，市图书馆、县文化中心、区三馆建成投入使用。积极推进金武大景区规划建设，加大圣容寺、三角城遗址、北海子景区等旅游资源的保护和开发力度，促进旅游与文化产业融合发展。加快永昌县城至圣容寺、河沟景区等旅游道路建设，大力发展乡村旅游，着力打造精品旅游线路，积极开发地方特色旅游产品。精心筹办好第三届骊靬文化国际旅游节和2014国际青少年生存训练营等节会活动，加大以骊靬文化、工业文化、红色文化和沙井文化为背景的文艺创作力度，提升文化软实力。
    加快金融服务业发展。优化金融生态环境，壮大资本实力，扩大信贷规模，建设多元化、多层次的现代金融体系，提高金融服务地方经济社会发展的质量和水平，全市各项贷款增长15%以上，金融机构向中小微企业发放贷款余额80亿元，同比增长40%。壮大公航旅等融资性担保公司实力，扩大城市建设投融资公司融资规模，加快组建开发区投资公司、交通投资公司和困难农户贷款政策性融资担保公司，做好建投企业债券发行工作。加快金川集团公司、金泥集团公司、三洋金源农牧公司、宇恒镍网公司等企业上市步伐，拓展直接融资渠道。推进地方金融机构发展，积极推行农村房屋产权、林权、设施农牧业产权、集体资产分配收益权、土地流转及承包经营权抵押融资，探索组建土地合作银行、村镇银行和农村金融互助社，全面提高农村金融运行质量和服务效率。
    （五）着力加强规划建设管理，切实加快新型城镇化步伐。积极探索符合金昌实际的新型城镇化道路，坚持高水平规划，高标准建设，高效能管理，不断提高城镇综合承载力和竞争力，争取全国文明城市创建成功。  
    提升规划层次。按照“生产空间集约高效、生活空间宜居适度、生态空间山清水秀”的总体要求，突出经济发展、扩大就业、吸纳人口、提高综合承载能力和公共服务水平，高水平编制新型城镇化规划、城市风貌规划、中心城区综合管网规划、公共场所林荫停车场规划、高新技术产业园区控制性详细规划，修编永昌县城、河西堡镇、朱王堡镇、水源镇总体规划，促进经济发展规划、土地利用规划和城市建设规划相互协调，科学有序、积极稳妥地向前推进城镇化。结合龙首新区、金水新区建设和老城区改造，着力提升建筑设计水平，做到建筑单体既符合规划要求、又彰显个性特征，建筑色彩和建筑风格协调搭配、自然和谐。进一步完善城市规划委员会审查制度，加大规划执法力度，做到无规划不审批、无规划不开发、无规划不建设，切实维护规划权威性，提高规划执行力。
    提速城市建设。围绕建设“活力镍都?戈壁绿城”，加快实施新区建设工程，重点实施市限价商品房、市公安系统综合业务用房等工程，开工建设新能源大厦、城市供水扩建等工程，市区热电联产管网工程、城市规划展览馆、市行政中心建成投入使用。加快实施街景风貌改造工程，加大三条主干道路街景风貌改造力度，加快西坡二期、28区城中村建设和白家嘴城中村改造进度，抓好金阿高速公路、车用天然气加气站项目建设，开工建设金昌至红沙岗一级公路。加快实施景观绿化工程，重点实施新华路金水新区景观带、龙泉景观带三期、国家矿山公园、龙首湖公园等城市绿化景观工程，全面完成金水湖段金川河东岸和上海路、南京路、新材料园区纵六路等绿化任务，让城市融入大自然，让居民望得见山、看得见水、记得住乡愁。加快实施县城和中心镇扩容提质工程，切实抓好永昌县城供水供热、中水利用、城区道路、312国道过城段改造、客运中心等公共基础设施建设项目，大力实施河西堡镇三年面貌大变样工程和重点小城镇镇区扩容提质项目，增强城镇产业承接能力，推进农业转移人口市民化。加强建筑施工安全生产管理，严厉打击超越资质范围承揽工程、违法分包转包工程等不法行为。
    提高管理水平。强化城市管理综合执法，推进城市管理服务网格化、精细化和智能化。坚持以治脏为重点，巩固国家卫生城市创建成果，加大背街小巷、居民小区等卫生盲点整治力度，营造干净整洁、舒适舒畅的卫生环境。坚持以治乱为重点，严肃查处乱堆乱放、乱停乱摆、乱贴乱画等不文明行为，下功夫整治城郊四村乱搭滥建现象，营造整洁优美、规范有序的市容环境。坚持以治差为重点，坚决取缔马路市场、流动摊点、店外经营等违章行为，切实加强农贸市场、商业网点和“五小”行业管理，营造整洁规范、和谐有序的市场环境。坚持以治堵为重点，通过建设电子监控系统、完善交通基础设施、规范司乘人员行为等，依法治理乱停车辆、乱穿马路、乱闯红灯、逆向行驶等违法违章行为，营造道路畅通、群众方便的交通环境。坚持以治污为重点，深入开展国家环保模范城市创建工作，积极倡导绿色低碳的生产生活方式，坚持脱硫脱硝脱氮并举、多污染物协同控制，重点实施市区污水处理工程升级改造及中水利用、开发区污水处理、河西堡污水处理等项目，营造用水安全、空气清新的生活环境。
    （六）着力促进改革开放，切实增强发展内生动力。认真贯彻落实十八届三中全会精神，加快推进重点领域和关键环节改革，建立充满活力、富有效率、促进科学发展的体制机制。
    加快改革步伐。稳步推进政府机构改革，分类推进事业单位改革，组建卫生和计划生育委员会，积极探索从事公益服务事业单位管办分离的有效实现形式，推动公办事业单位与行政主管部门理顺关系和去行政化。进一步明确政府基本职责和权力边界，切实做好为金川集团公司、八冶建设集团等优势骨干企业的服务，激活市场主体活力。加快推进以产权改革为重点的国有企业改革，着力支持金川集团公司深化产权改革，全面放开有色金属深加工领域，走产权多元化路子。逐步将市属国有企业改制为混合所有制企业，建立健全协调运转、有效制衡的公司法人治理结构，提高决策能力与管理水平。不断深化企业内部改革，建立完善管理人员能上能下、员工能进能出、收入能增能减的制度和投资经营责任制，合理确定并严格规范国有企业管理人员薪酬水平、职务待遇、职务消费、业务消费。积极推进城乡综合配套改革，着力消除依附在户籍制度上的公共服务和社会福利城乡差别，加快农业转移人口市民化进程，城镇化率达到68%。坚持量入为出、量力而行、尽力而为、收支平衡的原则，切实强化预算管理，规范预算执行，把政府性债务分门别类纳入全口径预算管理，严格政府举债程序，着力化解政府债务。建立跨年度预算平衡机制，形成预算透明、管理规范、动态监控、事权与支出责任相适应的现代财政制度。
    发展外向经济。紧紧抓住国家扩大内陆延边开放、推进“丝绸之路经济带”建设的重大政策机遇，坚持出口与进口并重、“引进来”与“走出去”并举，支持企业充分利用国际国内两个市场、两种资源，加强与“丝绸之路经济带”沿线各地各国的有效对接和密切联系，加快向西向北开放步伐。重点支持金川集团公司和八冶建设集团公司在中亚五国布局产业链，开展资源开发和工程承包，加快培育一批跨国经营集团。金昌海关和出入境检验检疫局年内开展报关报检业务，进一步提高经济外向度。以骊靬文化为桥梁和纽带，强化与意大利博拉、佩鲁贾等城市交流与合作。
    壮大非公实力。坚持权利平等、机会平等、规则平等，毫不动摇地鼓励、支持、引导非公有制经济发展，坚决废除各种不合理规定，全面消除各种隐性壁垒，保证各种所有制经济依法平等使用生产要素、公开公平公正参与市场竞争、同等受到法律保护。实行更加有利于实体经济发展的政策措施，加快推进优质中小企业培育计划、创新型中小企业成长计划、生产性服务业领域中小企业发展计划和金融支持中小微企业发展计划，鼓励引导中小微企业围绕工业园区、骨干企业、重点项目开展配套协作，向专精特新方向发展，新增规模以上工业企业5家。坚持“非禁即入”，积极推行商事登记“宽进严管”制度，实行注册资本认缴制，拓宽民间投资领域，促进民间投资较快增长。
    （七）着力发展社会事业，切实维护社会和谐安定。围绕学有优教、劳有多得、病有良医、老有善养、住有宜居“五有目标”，坚持把保障和改善民生作为政府一切工作的出发点和落脚点，努力为人民群众增进更多福祉。
    加快推进教育均衡发展。加快实施冶金学院二期和理工中专迁建等项目，深化产教融合、校企合作，促进职业教育与园区企业对接，力争把金昌职教城打造成全省高技能人才培训基地。推动各级各类教育协调发展，逐步缩小区域、城乡、校际差距，抓好市五中新建及永昌六中宿舍楼等项目，实施“教育四通云平台”网络建设和农村中小学信息化改造工程，提高教育质量，促进教育公平。着力推进“人才特区”建设，重点加强适应产业需求、紧贴产业转型升级的高层次急需紧缺人才和高素质技能型人才培养引进工作，为加快发展、转型跨越提供强有力的人才支撑。
    努力提高公共卫生服务水平。围绕创建国家健康城市，以“保健在家庭、小病在乡村、大病不出县”为目标，统筹推进医疗保障、医疗服务、药品供应和监管体制等领域综合改革，深化公立医院改革，进一步提升公立医院服务能力和管理水平。加快市人民医院迁建、市第一人民医院迁建、区中医院迁建、市妇幼保健站综合业务楼等项目建设进度，健全网络化城乡基层医疗服务体系，完善重特大医疗保障和救助制度，建立引导卫生人才下沉机制，为群众提供安全有效、方便价廉的公共卫生和基本医疗服务。坚持计划生育基本国策，启动实施“单独两孩”政策，促进人口长期均衡发展。加强公共体育设施建设，深入实施全民健身计划，积极发展竞技体育。
    大力推进科技创新。深入实施创新驱动发展战略，大力推进传统产业技术创新、战略性新兴产业科技成果转化、产业技术创新联盟发展、企业创新能力培育等“四大科技创新工程”，争取突破一批关键核心技术，培育一批创新型企业和高新技术企业。支持和引导园区和重点企业设立科技研发中心和推广机构，建立市级科技研发中心3个。加快甘肃有色金属新材料创新创业示范园建设，开发区科技孵化及检测中心建成投用。强化农业科技推广服务，建立农业科技示范点5个，示范面积5万亩。鼓励科技人员领办创办科技型企业，促进科技成果资本化、产业化。
    全力加强社会治理。组织开展安全生产大排查、矛盾纠纷大调处、交通安全大整顿、消防隐患大排除和社会治安大整治等社会治理“五大专项行动”，继续推进“平安金昌”建设，不断完善社会治安防控体系，切实加强社会稳定风险评估工作，大力扫除“黄赌毒”等社会丑恶现象，严厉打击各种违法犯罪，确保人民安居乐业、社会安定有序。加快应急指挥平台建设，完善自然灾害、事故灾难、公共卫生和社会安全等突发公共事件应急体系建设。深入创建双拥模范城“七连冠”，巩固军政军民团结，促进军民融合式发展。深入推进“和谐寺观教堂”创建工作，大力加强“两个共同”示范区建设，促进各族群众和睦相处、和衷共济、和谐发展。认真做好第三次全国经济普查工作，继续做好防震减灾、气象服务、外事侨务等工作，扎实做好对口援藏工作。
    集中精力办好民生实事。按照择重择急、尽力而为、当年办结的原则，集中力量办好10件民生实事。1.完成农村初中校舍改造工程2.5万平米，配套完善10个乡镇高标准中心幼儿园。2.实施农民工文化服务工程，全市公共文化服务场所全部免费开放，为进城务工人员享受公共文化服务提供便利条件。3.积极开展创业型城市创建工作，完成职业技能培训6500人，创业能力培训1400人。新增城镇就业1.2万人、小额担保贷款1亿元；普通高校应届毕业生就业率达90%以上；扶持帮助500名残疾人实现就业和自主创业。4.城乡居民医疗保险财政补助标准由280元提高到320元，人均筹资标准达到400元。5.积极推行城乡低保三年并轨计划，城乡低保标准提高15%；为特别困难残疾人发放生活补贴。6.完成16所标准化村卫生室和7所乡镇卫生院附属用房建设任务。7.实施幸福银发夕阳工程，建设40个农村互助老人幸福院，4个老年人日间照料中心；提高城乡居民社会养老保险基础养老金补助标准。8.新建保障性住房13076套，其中廉租房184套、公租房7458套、城市棚户区改造5434套。9.新建“一村一场”农民体育健身工程10个，体育惠民工程2个，乡镇农民体育健身提升工程1个。10.强化食品药品监管，推行食品药品电子监管工作，实施零售药店便民电子查询和食品检验检测中心工程，放心粮、放心油、放心肉、放心菜和放心奶达标示范店各达8家以上。
    （八）着力加强政府自身建设，切实提高执行力、凝聚力和公信力。全面完成今年的目标任务，我们必须不断加强政府自身建设，扎实开展群众路线教育实践活动，进一步严肃政风政纪，提高行政效能。
    务实高效提高执行力。全面落实中央和省、市委改进工作作风、密切联系群众的规定，坚决克服官僚主义、形式主义、享乐主义和奢靡之风，深入推进效能风暴行动，继续大力推行“一线工作法”和“三三制”工作方法，下功夫精简各类会议，积极倡导开短会、写短文、讲短话，把时间和精力用在抓发展、惠民生、促和谐上。按照中央关于改进地方党政领导班子和领导干部政绩考核工作的要求，进一步健全完善“六大考核激励机制”，强化任期内举债情况考核、审计和责任追究，坚决不搞华而不实、劳民伤财的“形象工程”，急功近利的“半拉子工程”，沽名钓誉的“政绩工程”，使政府工作经得起实践、人民、历史的检验。扎实开展政策执行情况监督检查，防止政策悬空、资金沉淀、权力寻租，以更加有力的举措，营造风清气正、干事创业的政治生态环境。
    勤政为民增强凝聚力。坚持为公用权、为民用权，切实增强公仆意识，恪守为民之责、善谋富民之策、多办利民之事，真正做到为人民服务，对人民负责，受人民监督，让人民满意。认真开展“四减五制三集中”活动，下功夫减行政审批事项、环节、时限、费用，推行窗口受理制、首问负责制、一次性告知制、限时办结制、责任追究制，实现部门行政审批事项向一个科室集中，为民服务事项向市政府政务服务中心集中，招商引资服务事项向开发区综合服务中心集中，向市场、社会、企业和公民放权松绑，做到办事程序最简、时限最短、效率最高、服务最优。加强电子政务建设，加大政府信息公开力度，加快公共资源电子交易系统建设，强化市长热线、市长信箱等网络问政工作，及时回应群众关切，解决群众诉求。
    廉洁从政提升公信力。自觉接受人大工作监督、法律监督和政协民主监督、社会舆论监督，充分听取各民主党派、工商联、无党派人士和各人民团体的意见建议，认真办理人大代表建议、政协委员提案。深入推进惩治和预防腐败体系建设，严格落实党风廉政建设责任制，认真贯彻《党政机关厉行节约反对浪费条例》，坚决杜绝在经费管理、差旅活动、公务接待、公务用车、会议活动、办公用房、资源节约等方面的奢侈浪费现象，严厉查处公务支出中的奢靡腐败行为，真正做到勤俭办一切事情。突出法治政府建设，积极推进“六五”普法工作，加快电子监察系统建设，加大行政问责力度，依法公开权力运行流程，把权力关进制度的笼子里，推进决策公开、管理公开、服务公开、结果公开，做到依法办事、依法决策、依法处理问题。这里我代表市人民政府向全市人民承诺：抓政风建设，首先从市政府做起，要求别人做到的自己首先做到，要求别人不做的自己坚决不做！
    各位代表，梦想孕育奇迹，万事出自艰辛。让我们在市委的坚强领导下，在市人大、市政协的监督支持下，解放思想，改革创新，凝聚力量，攻坚克难，在建设繁荣文明和谐宜居现代金昌的征程中，谱写中国梦金昌美好新篇章！
